# High Expression of PDK4 Could Play a Potentially Protective Role by Attenuating Oxidative Stress after Subarachnoid Hemorrhage

**DOI:** 10.3390/jcm11143974

**Published:** 2022-07-08

**Authors:** Xuan Gao, Yong-Yue Gao, Ling-Yun Wu, Zheng Peng, Xun-Zhi Liu, Xiang-Xin Chen, Sen Gao, Hua-Sheng Zhang, Yue Lu, Chun-Hua Hang, Zong Zhuang, Wei Li

**Affiliations:** 1 Department of Neurosurgery, Nanjing Drum Tower Hospital, The Affiliated Hospital of Nanjing University Medical School, Nanjing 210008, China; xuan_gao@126.com (X.G.); tallergao@163.com (Y.-Y.G.); dr.wulingyun@gmail.com (L.-Y.W.); neurosurgery_pz@163.com (Z.P.); 15105197877@163.com (X.-Z.L.); xiangxin.chen@foxmail.com (X.-X.C.); sen_gao@outlook.com (S.G.); njuzhs@163.com (H.-S.Z.); luyue120@nju.edu.cn (Y.L.); hang_neurosurgery@163.com (C.-H.H.); 2 Department of Neurosurgery, Tianjin Huanhu Hospital, Tianjin 300300, China

**Keywords:** PDK, PDH, pyruvate, oxidative stress, SAH, apoptosis

## Abstract

Pyruvate dehydrogenase (PDH), a key enzyme on the mitochondrial outer membrane, has been found to decrease activity notably in early brain injury (EBI) after subarachnoid hemorrhage (SAH). It has been demonstrated that PDH is associated with the production of reactive oxygen species (ROS) and apoptosis. Hence, in this study, we aimed to determine the cause of the decreased PDH activity and explore the potential role of PDH in EBI. We investigated the expression changes of PDH and pyruvate dehydrogenase kinase (PDK) in vivo and in vitro. Then, we explored the possible effects of PDH and ROS after SAH. The results showed that early overexpression of PDK4 promoted the phosphorylation of PDH, inhibited PDH activity, and may play a protective role after SAH in vivo and in vitro. Finally, we investigated the levels of PDK4 and pyruvate, which accumulated due to decreased PDH activity, in the cerebrospinal fluid (CSF) of 34 patients with SAH. Statistical analysis revealed that PDK4 and pyruvate expression was elevated in the CSF of SAH patients compared with that of controls, and this high expression correlated with the degree of neurological impairment and long-term outcome. Taken together, the results show that PDK4 has the potential to serve as a new therapeutic target and biomarker for assisting in the diagnosis of SAH severity and prediction of recovery.

## 1. Introduction

Subarachnoid hemorrhage (SAH) is a central nervous system disease with high mortality and morbidity [1,2,3]. Early brain injury (EBI) is the most common cause of severe neurological impairment and unfavorable prognosis in SAH patients [4,5]. EBI occurs within 72 h after SAH and includes various mechanisms: blood-brain barrier disruption, inflammation, oxidative stress, cell death, and mitochondrial dysfunction [6]. Although many treatments have been designed, their translation into clinical practice still fails. We need to make greater efforts to explore the pathology of EBI and find new therapeutic targets.

Pyruvate dehydrogenase (PDH), located in the outer mitochondrial membrane, is a key regulatory enzyme in energy metabolism. It catalyzes the conversion of pyruvate to acetyl-coenzyme A (acetyl-CoA) [7,8,9]. Pyruvate dehydrogenase kinase (PDK) is also located in the outer mitochondrial membrane and can negatively regulate PDH activity by phosphorylating one of its subunits. PDK has four known tissue-specific isozymes that share 70% DNA sequences [10]. PDK1, the largest of the four isozymes, contains 436 amino acid residues. PDK2, PDK3, and PDK4 are composed of 407, 406, and 411 amino acid residues, respectively. Furthermore, various isozymes play different roles. PDK1 is expressed mostly in cardiomyocytes; PDK2 is expressed widely except in spleen and lung; PDK3 is expressed mainly in spermary; and PDK4 is mainly expressed in muscle and in the brain, which consume a great amount of oxygen [11,12]. The effects of PDK and PDH on metabolism play an important role in many pathological processes. In a study of cancer, it was demonstrated that the overexpression of PDK1 results in a decrease in the activity of PDH, affects the metabolic state of cancer cells, and promotes the growth, survival, and invasion of cancer cells [13,14,15]. In addition, some studies have demonstrated that activated PDH promotes the production of ROS, induces mitochondrial outer membrane permeabilization (MOMP), and leads to apoptosis. In recent years, it has been demonstrated that pyruvate accumulates as a result of suppression of PDH activity and may scavenge ROS and protective cells [16,17,18].

After the establishment of the SAH model, it was found that the activity of PDH decreases obviously, oxidative metabolism is disrupted, and pyruvate levels are increased [19,20,21]. However, the reason for and likely consequences of the PDH activity decrease remain unclear. Thus, we assessed the expression of PDK and PDH in vivo and in vitro after SAH by Western blotting (WB) and real-time quantitative polymerase chain reaction (qPCR). The levels of PDK and pyruvate in the CSF of patients with SAH were assessed, and we analyzed whether PDK or pyruvate can be a predictor of severe injuries and/or unfavorable outcomes.

## 2. Method and Materials

### 2.1. Rat Model

A total of 75 healthy adult male Sprague–Dawley rats weighing 230–320 g were purchased from Animal Core Facility of Nanjing Medical University. All experiments were carried out under the Guide for the Care and Use of Laboratory Animals published by NIH and approved by the Experimental Animal Ethics Committee of Nanjing Drum Tower, approval number: 2020AE02015. All animal studies are reported in compliance with the ARRIVE guidelines [22,23].

The endovascular perforation SAH model was performed as previously described [24,25,26]. The endovascular perforation surgery was performed by the same experimenter (X.G.). Briefly, rats were first anaesthetized by isoflurane inhalation (RWD Life Science, Shenzhen). After sharp and blunt separation, a marked 6-0 filament was inserted to the middle cerebral artery (MCA) through the internal carotid artery (ICA). We punctured the bifurcation of the anterior and middle cerebral arteries in SAH group. In sham group, the filament was pulled out without puncturing the artery.

The neurological score was blindly evaluated by two independent observers by using a Modified Garcia scale score 24 h post-SAH [26,27]. Rats with a Modified Garcia scale score ≤ 6 or ≥15 were excluded to prevent the interference of dying models and failure SAH models. 

### 2.2. Primary Neuron Culture

For the neuron culture, the cortex was obtained from rats at embryonic day 13–15 as we previously reported [24,25,26]. In brief, after removing leptomeninges, the cerebral cortex was digested with Trypsin. Then we used fetal bovine serum to stop the digestion and repeatedly triturated the neuron suspension. After filtration and centrifugation, the remaining neurons were seeded in poly-D-lysine-coated plates with neurobasal medium and incubated at 37 °C and 5% CO_2_. The medium contained 0.5 mM GlutaMax (Gibco Company, New York, NY, USA) and 2% B27 supplement (Gibco Company, USA). We would replace the medium at 4 h, 3 d, 5 d, and 7 d following seeding. For the in vitro SAH model, neurons were treated with hemoglobin (Hb, Sigma, Saint Louis, MO, USA) at a concentration of 25 μM for 24 h. Primary neurons were randomly assigned to different groups.

### 2.3. Patient Population

Patients with SAH were recruited from the Department of Neurosurgery of the Nanjing Drum Tower Hospital between April 2020 and April 2021. A diagnosis of SAH required a positive cranial CT scan and bloody cerebrospinal fluid. For all SAH subjects, the inclusion criteria were:

(1) patient age between 16 and 70 years;
(2) signed consent from the subject or next of kin; and
(3) the patient had no contraindications to lumbar puncture.

The exclusion criteria were:

(1) failure to meet the inclusion criteria or unfit for the experiment as determined by the responsible doctor;
(2) presence of severe cardiac insufficiency, renal dysfunction, diabetes or other systemic diseases; and
(3) a history of traumatic brain injury, severe cerebral edema or hydrocephaly.

As a control group, knee arthritis subjects who needed surgery were recruited. For control subjects, the inclusion criteria were:

(1) patient age between 16 and 70 years;
(2) no current or pre-existing brain injuries, neurological diseases, or bleeding disorders, and subjects who needed surgery for knee arthritis; and
(3) signed consent from the subject or next of kin.

Ultimately, 34 patients with SAH and 6 controls were included. Patient demographics are shown in Table 1. The study was approved by the Ethics Committee of Drum Tower Hospital (No. 2020-041-01) and registered in the Chinese Clinical Trial Registry (ChiCTR2100042986). Written informed consent was obtained from each participant.

### 2.4. Sample Collection

Cerebrospinal fluid (CSF) samples were collected within 72 h after SAH following the consensus protocol for the standardization of cerebrospinal fluid collection [28]. The cerebrospinal fluid was retained as samples and centrifuged at 2000× *g* for 15 min at 4 °C to pellet cellular bodies and debris. All samples were stored at −80 °C.

### 2.5. Experimental Design

**Figure 1:**
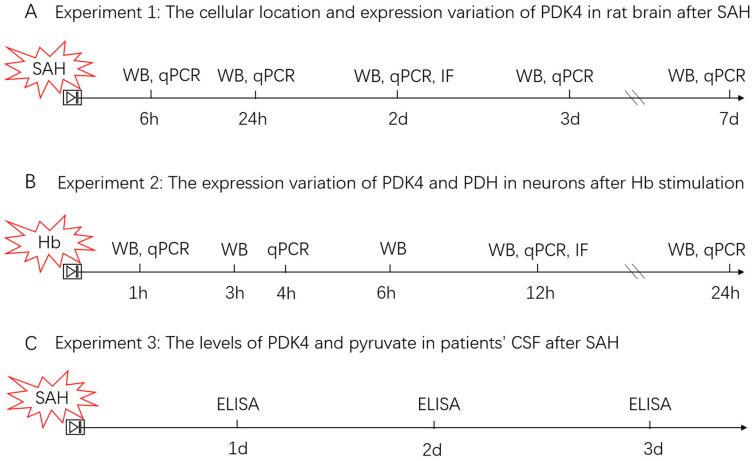
The experimental designs and groups. The experimental designs in vivo (**A**), in vitro (**B**), and in SAH patients’ CSF (**C**). WB, western blotting; qPCR, quantitative real-time polymerase chain reaction; IF, immunofluorescence staining; h, hours; d, days; SAH, subarachnoid hemorrhage; Hb, hemoglobin; CSF, cerebrospinal fluid.

The main experimental protocols are described as follows (Figure 1):

#### 2.5.1. Experiment 1

To examine the expression variation of PDKs and PDH in rat brain after SAH, a total of 50 rats were randomly assigned to six groups: sham (*n* = 5), 6 h post-SAH (*n* = 9), 24 h post-SAH (*n* = 9), 2 days post-SAH (*n* = 9), 3 days post-SAH (*n* = 9), and 7 days post-SAH (*n* = 9). Five rats from each group were sacrificed randomly for WB and qPCR. In stage 2, 15 rats were randomly allocated into two groups: the sham group (*n* = 6) and the SAH group (2 days) (*n* = 9). Six rats from each group were selected randomly for immunofluorescence staining.

#### 2.5.2. Experiment 2

To examine the expression variation of PDKs and PDH in cultured neurons after Hb stimulation, Hb from bovine erythrocytes (Sigma, USA) was dissolved in complete culture medium at a concentration of 25 μmol/L. Cultured neurons were randomly assigned to six groups (the control group and Hb groups (1 h, 3 h, 6 h, 12 h, 24 h) (*n* = 5 each)) for WB and five groups (the control group and Hb groups (1 h, 4 h, 12 h, 24 h) (*n* = 5 each)) for qPCR. Finally, the control group and 12 h post-Hb group were used for immunofluorescence staining and TUNEL staining.

#### 2.5.3. Experiment 3

To examine the levels of PDK4 and pyruvate in the CSF of patients with SAH, CSF samples were collected from 34 SAH patients and 6 controls for ELISA.

### 2.6. Western Blot 

The brains were removed and the basal cortex tissues were collected and washed. Neurons cultured in six-well plates were washed with phosphate-buffered saline (PBS). Cortex tissues and neurons were lysed with RIPA buffer (Thermo Scientific, Waltham, MA, USA) with protease inhibitor (Roche, Basel, Switzerland) and phosphatase inhibitor (Thermo Fisher Scientific). A bicinchoninic acid protein assay kit (Beyotime, Shanghai, China) was used to quantify the amounts of protein. The same amount of protein was resolved by SDA-PAGE (EpiZyme Scientific, Cambridge, MA, USA) and transferred to polyvinylidene difluoride membranes (Millipore, Burlington, MA, USA). We used 2% BSA to block the membranes for 2 h at room temperature. After incubation with diluted primary antibody overnight at 4 °C, the membranes were washed 3 times for 10 min with PBS and Tween 20. The antibodies were as follows: anti-PDK1 (1:1000, 3820, Cell Signaling Technology, Danvers, MA, USA), anti-PDK2 (1:1000, 68164, Abcam, Cambridge, UK), anti-PDK3 (1:1000, 154549, Abcam), anti-PDK4 (1:1000, 89295, Abcam), anti-PDH (1:1000, 3205, Cell Signaling Technology), anti-phospho-PDH (S293) (1:1000, 177461, Abcam), anti-ASK1 (1:1000, 45178, Abcam), anti-phospho-ASK1 (Thr845) (1:1000, 3765, Cell Signaling Technology), anti-P38 (1:1000, 9212, Cell Signaling Technology), anti-phospho-P38 (Thr180/Tyr182) (1:1000, 9211, Cell Signaling Technology), anti-BAX (1:1000, 2772, Cell Signaling Technology), anti-BCL2 (1:1000, 32124, Abcam), anti-caspase3 (1:1000, 9662, Cell Signaling Technology), anti-cleaved-caspase3 (Asp175) (1:1000, 9664, Cell Signaling Technology), and anti-GAPDH (1:5000, AP0066, Bioworld, Irving, TX, USA). The membranes were incubated with horseradish peroxidase (HRP)-conjugated secondary antibody (Bioworld) for 1 h at room temperature. Finally, the bands were detected by Immobilon Western Chemiluminescent HRP Substrate (Millipore Sigma, Burlington, MA, USA). Images were analyzed with ImageJ software (National Institutes of Health, Bethesda, MD, USA) and normalized against GAPDH. 

### 2.7. Real-Time PCR

The total RNA of basal cortex tissues and neurons was extracted by TRIzol reagent (Invitrogen, Carlsbad, CA, USA) according to the manufacturer’s instructions. cDNA was reverse transcribed from mRNA with a reverse transcription mix (Vazyme, Nanjing, China) after removal of genomic DNA. qPCR was performed by using a PCR system (Applied Biosystems, Waltham, MA, USA) with a SYBER Green mix (Roche, Switzerland). The primers used in qPCR are listed in Table 2. The results were analyzed with the 2^−ΔΔCt^ method and normalized against GAPDH too.

### 2.8. Immunofluorescence Staining

Brain tissue was postfixed in 4% paraformaldehyde, dehydrated with sucrose solution and sliced to 10 μm. Cultured neurons were fixed with 4% paraformaldehyde. Then they were permeabilized with 0.3% Triton X-100, and blocked with immunostaining blocking solution (Epizyme, Shanghai, China). After incubation with diluted primary antibody overnight at 4 ℃, the membranes were washed 3 times for 10 min with PBS and Tween 20. The antibodies were as follows: anti-PDK4 (1:200, 89295, Abcam), anti-NeuN (1:200, 26975-1-AP, Proteintech, Rosemont, IL, USA), anti-Iba1 (1:200, 5076, Abcam), anti-GFAP (1:200, 4648, Abcam). The next day, they were incubated with corresponding secondary antibodies: anti-rabbit Alex Fluor 488-conjugated secondary antibody (1:200, A11008, Invitrogen), anti-rabbit Alexa Fluor 594-conjugated secondary antibody (1:200, A32754, Invitrogen), anti-mice Alexa Fluor 594-conjugated secondary antibody (1:200, A32740, Invitrogen), anti-mice Alexa Fluor 488-conjugated secondary antibody (1:200, A32723, Invitrogen). Immunofluorescence images were captured by using the microscope (ZEISS, HB050, Berlin, Germany) and analyzed with ImageJ.

### 2.9. Enzyme Linked Immunosorbent Assay

The levels of PDK4 were determined by an ELISA kit (abx252933, Abbexa, Cambridge, UK) according to the manufacturer’s instruction. In brief, CSF and standard samples were loaded in to the 96-well plate incubated with PDK4 antibody. After incubation of detection reagent A and B, the TMB substrate and stop solution were added to every well. Finally, the results were detected at OD 450 nm in a microplate reader (Tecan, Männedorf, Switzerland). The levels of PDK4 were calculated according to the standard curve.

### 2.10. Pyruvate Assay

The levels of pyruvate were detected by a pyruvate assay kit (#K609-100, Biovision, Milpitas, CA, USA) according to the manufacturer’s instructions. Briefly, after brain tissue and cultured neurons were extracted, samples were homogenized. Subsequently, the supernatant was collected after centrifugation at 10,000× *g* for 10 min at 4 °C. Finally, the fluorescence was detected at an Ex/Em of 435/590 nm in a microplate reader (Tecan, Switzerland). A preliminary experiment in which color (OD 570 nm) was used to calculate the results was essential to determine the appropriate loading volume. The pyruvate contents were calculated according to the manufacturer’s instructions.

### 2.11. Terminal Deoxynucleotidyl Transferase–Mediated dUTP Nick End Labeling

TUNEL staining was performed on frozen brain sections and cultured neurons with a TUNEL detection kit (Beyotime, China) according to the manufacturer’s instructions. After incubation with a primary antibody against NeuN (1:200, 26975-1-AP, Proteintech) at 4 °C overnight, the TUNEL reaction mixture were loaded into every section for 1 h at 37 °C. The images were captured by using the microscope (ZEISS, HB050, Germany) and analyzed with ImageJ.

### 2.12. Modified Garcia Scale

We used the Modified Garcia scale to assess the functional defects at 1 day post-SAH by two observers who were blind to groups [26,29]. It contains 6 tests covering spontaneous activity and movement of the four limbs, forepaw outstretching, climbing, body proprioception, and response to whisker stimulation.

### 2.13. Statistical Analysis

Glasgow Coma Scale (GCS) scores 1–3 days after SAH were categorized as severe (GCS score < 12) or mild (GCS score 12–15) injury for each subject.

Hunt–Hess Scale scores were acquired at the onset of SAH, and the lumbar puncture was performed on day 1–3 after SAH. The subjects were divided into two groups: those with Hunt–Hess scores of 1–2 and those with Hunt–Hess scores of 3–4.

Glasgow Outcome Scale (GOS) scores at 3 months were compared with those at 1–3 days after SAH to sort subjects into an improved group and an unchanged group for ease of presentation and interpretation of results. Subjects whose initial GOS scores and GOS scores after 3 months were both 5 were excluded.

Statistical analysis was performed by using Prism 8.01 (GraphPad Software, San Diego, CA, USA). A two-tailed unpaired Student’s *t*-test was used to compare two experimental groups after normality and lognormality tests were satisfied. One-way ANOVA followed by Tukey’s test was performed to assess differences between more than two groups. Two-way ANOVA was used to assess the interaction effects of treatments and time courses. *p* < 0.05 was considered statistically significant. All data are expressed as the means ± SD.

## 3. Results

### 3.1. Mortality and Exclusion

No rats died in the sham group, and the overall mortality of the SAH group was 18.5% (10/54). According to the Modified Garcia scores at 1 day post-SAH, a total of 13 rats were excluded from this study (Table 3).

### 3.2. The Expression of PDK4 but Not PDH Was Elevated in Neurons after SAH in Rats

**Figure 2:**
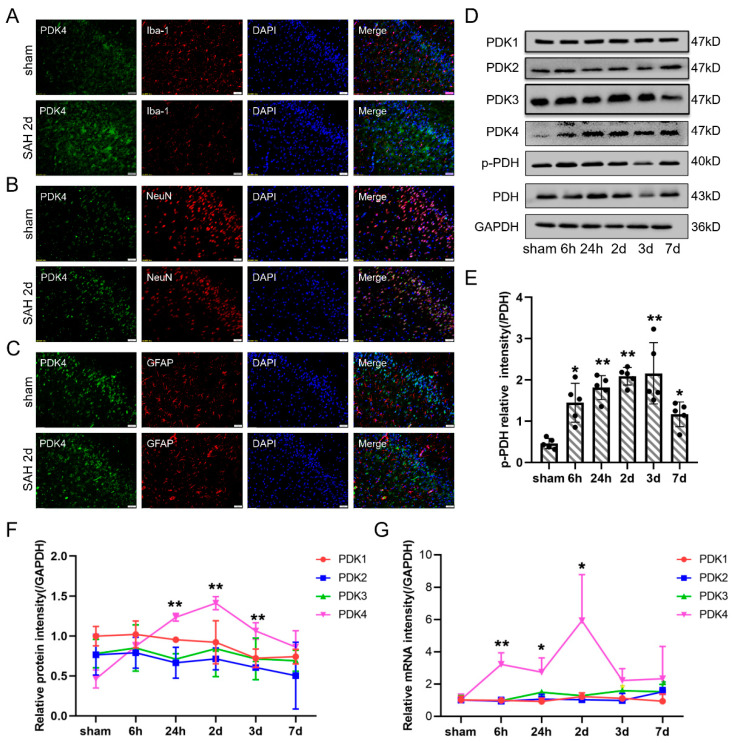
The expression and cellular distribution of PDKs in rat brains after SAH. Representative immunofluorescence staining of PDK4, Iba1 (a microglial marker) (**A**), NeuN (a neuronal marker) (**B**), and GFAP (an astrocytic marker) (**C**) in the cortex of the right temporal lobe after SAH (PDK4, green; NeuN, Iba1, and GFAP, red; and DAPI, blue). (**D**) Representative bands of PDK1, PDK2, PDK3, PDK4, PDH, and p-PDH expression in the cortex at each time point (0, 6, and 24 h and 2, 3, and 7 d) after SAH. (**E**) Quantitative analysis of Western blot results showed that the ratio of p-PDH/PDH was significantly increased after SAH. (**F**,**G**) Quantitative analysis of Western blot and qPCR results showed that variation of PDKs protein and mRNA levels after SAH. Bars represent the means ± SD. * *p* < 0.05; ** *p* < 0.01; vs. sham (*n* = 5 in each group). Bar = 50 μm.

To determine the expression of PDKs and PDH at different time points after SAH, rat brain tissues were obtained and analyzed by Western blot and qPCR (Figure 2D–G). PDK4 was significantly elevated 24 h after SAH and peaked at 2 days. There were no significant differences in the expression of PDK1, PDK2, or PDK3 after SAH. Consistent with PDK4, p-PDH reached peak expression at 2 and 3 days after SAH, while the expression of PDH showed little change (Figure 2D,E). Immunofluorescence staining showed that PDK4 was highly expressed in neurons (Figure 2B), expressed at a low level in microglia (Figure 2A), and barely expressed in astrocytes (Figure 2C). The results verified that PDK4 was overexpressed mainly in neurons after SAH.

### 3.3. Neuronal Apoptosis Participated in the Pathology of EBI In Vivo

**Figure 3:**
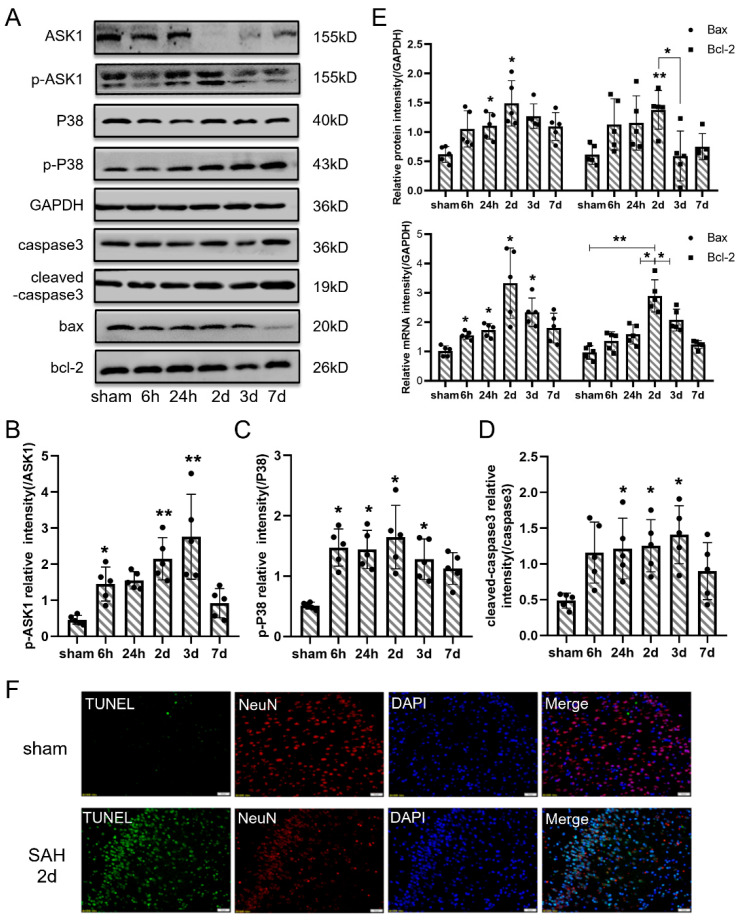
The apoptosis pathway was activated after SAH. (**A**) Representative bands of ASK1, p-ASK1, P38, p-P38, caspase3, cleaved-caspase3, Bax, and Bcl-2 expression in the cortex at each time point after SAH. (**B**–**D**) Quantitative analysis of Western blot results showed that the ratio of p-ASK1/ASK1, p-P38/P38 and cleaved-caspase3/caspase3 were significantly increased after SAH. (**E**) Quantitative analysis of Western blot and qPCR results showed that the protein and mRNA levels of Bax and Bcl-2 were increased after SAH. (**F**) Representative TUNEL staining in the cortex of right temporal lobe after SAH (TUNEL, green; NeuN, red; DAPI, blue). Bars represent the means ± SD. * *p* < 0.05; ** *p* < 0.01; vs. sham (*n* = 5 in each group). Bar = 50 μm.

To better understand the effect of overexpressed PDK4 after SAH, apoptosis and related agents were evaluated (Figure 3). The protein levels of p-ASK1 and p-p38 were significantly increased, and cleaved caspase3 accumulated after SAH (Figure 3A–D). In addition, the levels of Bax and Bcl-2 were upregulated at both the protein and mRNA levels 2 days after SAH (Figure 3E). Finally, TUNEL staining confirmed that neuronal apoptosis was significantly aggravated after SAH (Figure 3F). These results reflected that overexpressed PDK4 may be related to neuronal apoptosis after SAH.

### 3.4. Hb Exposure Also Induced the Overexpression of PDK4 in Neurons In Vitro

**Figure 4:**
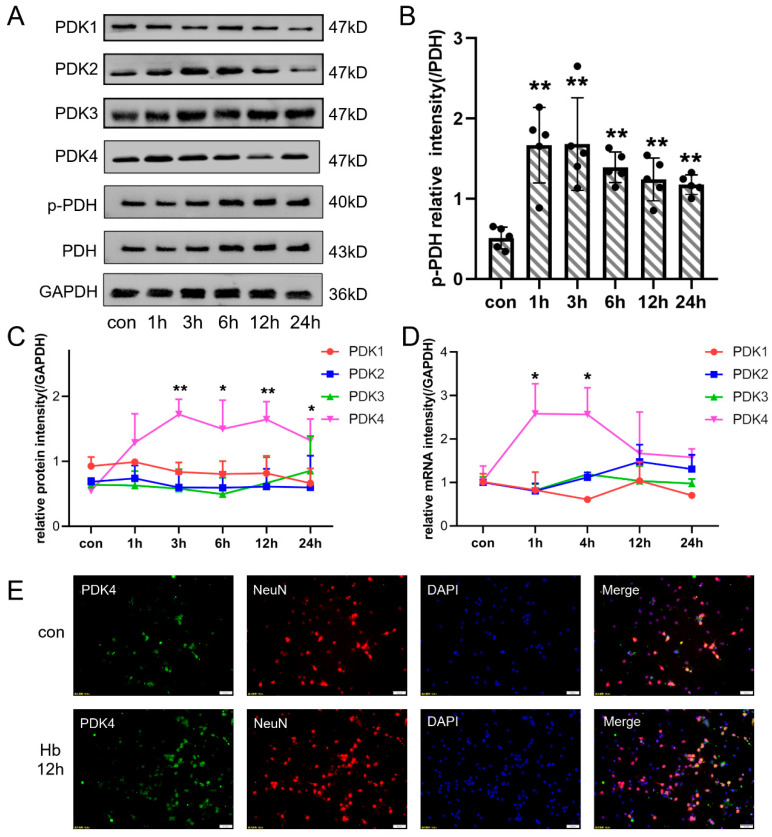
The expression of PDKs in cultured primary neurons after Hb stimulation. (**A**) Representative bands of PDK1, PDK2, PDK3, PDK4, PDH, and p-PDH expression at each time point (0, 1, 3, 6, 12, and 24 h) after Hb stimulation. (**B**) Quantitative analysis of Western blot results showed that the ratio of p-PDH/PDH was significantly increased after Hb stimulation. (**C**,**D**) Quantitative analysis of Western blot and qPCR results showed that variation of PDKs protein at each time point (0, 1, 3, 6, 12, and 24 h) and mRNA at each time point (0, 1, 4, 12, and 24 h) levels after Hb stimulation. (**E**) Representative immunofluorescence staining for PDK4 and NeuN (a neuronal marker) in cultured primary neurons after Hb stimulation (PDK4, green; NeuN, red; DAPI, blue). Bars represent the means ± SD. * *p* < 0.05; ** *p* < 0.01 vs. con (*n* = 5 in each group). Bar = 50 μm.

We constructed an in vitro SAH model by Hb stimulation and performed Western blotting and qPCR to assess the protein and mRNA expression of related genes. As shown in Figure 4C,D, the levels of PDK4 protein and mRNA were increased after Hb exposure and peaked at 3 h. The levels of PDK1, PDK2, and PDK3 were not different after Hb stimulation. Similar to the change in rats after SAH, PDH expression was not significantly altered after Hb stimulation, but p-PDH expression reached a peak at 1 h in neurons after Hb stimulation (Figure 4A,B). Immunofluorescence staining also showed that PDK4 was overexpressed in neurons after Hb stimulation (Figure 4E).

### 3.5. Hb Exposure Activated the Pathway of Neuronal Apoptosis In Vitro

**Figure 5:**
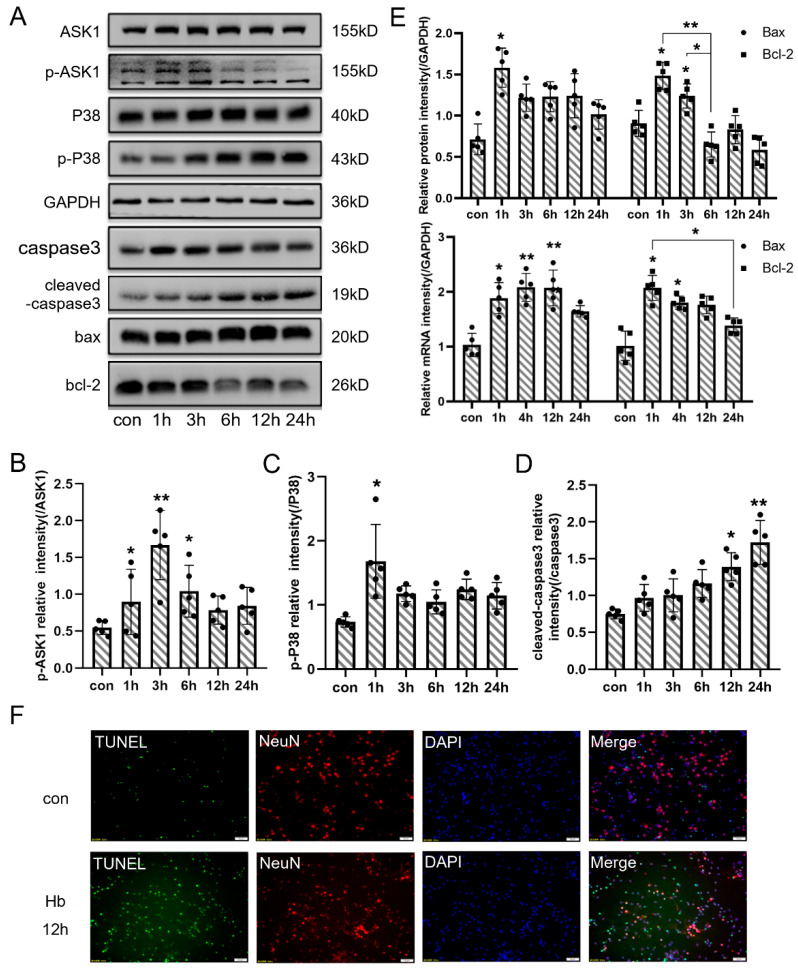
The neuronal apoptosis pathway was activated after Hb stimulation. (**A**) Representative bands of ASK1, p-ASK1, P38, p-P38, caspase3, cleaved-caspase3, Bax, and Bcl-2 expression in cultured primary neurons at each time point after Hb stimulation. (**B**–**D**) Quantitative analysis of Western blot results showed that the ratio of p-ASK1/ASK1, p-P38/P38 and cleaved-caspase3/caspase3 were significantly increased after Hb stimulation. (**E**) Quantitative analysis of Western blot and qPCR results showed that the protein and mRNA levels of Bax and Bcl-2 were increased after Hb stimulation. (**F**) Representative TUNEL staining in cultured primary neurons after Hb stimulation (TUNEL, green; NeuN, red; DAPI, blue). Bars represent the means ± SD. * *p* < 0.05; ** *p* < 0.01 vs. con (*n* = 5 in each group). Bar = 50 μm.

To verify that the pathological processes of the SAH in vitro model were consistent with those of the SAH in vivo model, apoptosis and related agents were assessed and found to be elevated in vitro (Figure 4). The levels of p-ASK1, p-p38 and cleaved caspase were obviously increased in neurons after Hb stimulation (Figure 5A–D). In addition, the protein and mRNA levels of Bax and Bcl-2 increased and peaked at 3 h in neurons after Hb stimulation (Figure 5E). TUNEL staining also reflected that neuronal apoptosis significantly increased after Hb stimulation (Figure 5F). These results indicated that neuronal apoptosis was involved in the pathological processes of the in vivo an in vitro SAH models.

### 3.6. The Expression of PDK4 Was Elevated in the CSF of SAH Patients

**Figure 6:**
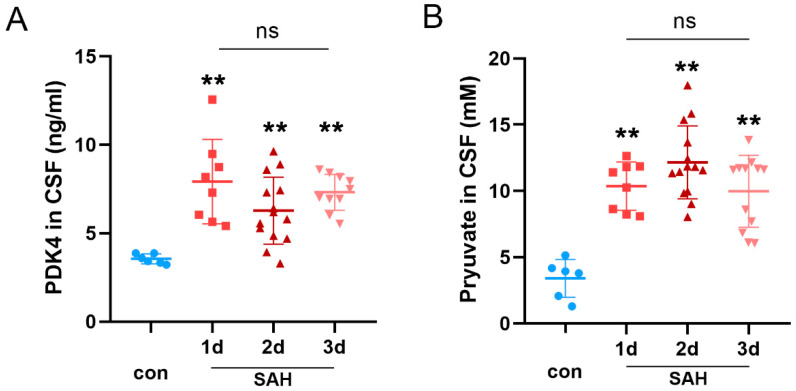
Scatter plots of the levels of PDK4 and pyruvate in controls and patients with SAH. Quantitative analysis of ELISA results showed that the levels of PDK4 (**A**) and pyruvate (**B**) were obviously increased in the CSF of patients 1–3 days after SAH compared with controls. Bars represent the means ± SD. ** *p* < 0.01 vs. con (*n* = 6 in the con group; *n* = 8, 14 and 10 in the groups on days 1, 2 and 3 after SAH, respectively).

As shown in Figure 6, the levels of PDK4 and pyruvate were significantly elevated in the CSF of SAH patients after 1–3 days compared with controls (both *p* < 0.0001). There was no difference in PDK4 and pyruvate levels each day after SAH. Two maximum values of PDK4 at 3 days in the SAH group were removed.

### 3.7. Different Levels of PDK4 and Pyruvate Were Associated with the Neurological Function Scores of SAH Patients

**Figure 7:**
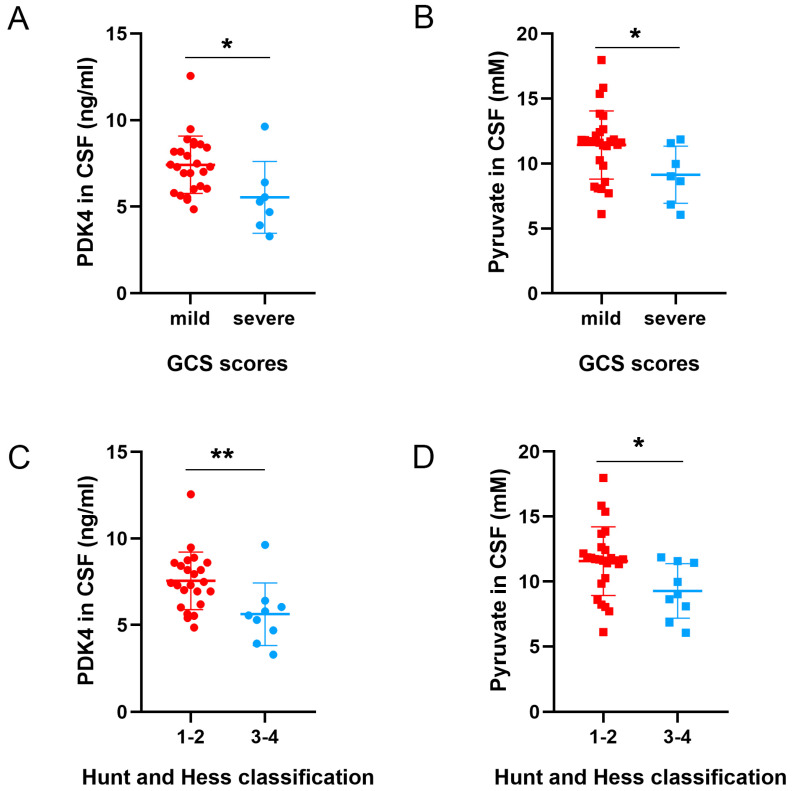
Scatter plots of the levels of PDK4 and pyruvate sorted by injury category. Unpaired two-tailed *t*-tests indicated that higher expression of PDK4 and pyruvate was significantly associated with mild neurological injury 1–3 days after SAH (**A**–**D**). Bars represent the means ± SD. * *p* < 0.05; ** *p* < 0.01 vs. the indicated groups (*n* = 7 in the severe injury group (GCS score < 12), *n* = 25 in the mild injury group (GCS score 12–15), *n* = 9 in the Hunt–Hess 3–4 group and *n* = 23 in the Hunt–Hess 1–2 group).

To determine whether the levels of PDK4 and pyruvate correlated with neurological injury, we grouped study participants by injury category: GCS scores < 12, severe injury; GCS scores 12–15, mild injury; Hunt–Hess scores 1–2; and Hunt–Hess scores 3–4. We detected significantly higher levels of PDK4 (Figure 7A,C) and pyruvate (Figure 7B,D) in the CSF of SAH patients with mild injury (*p* = 0.0176 and *p* = 0.0414) and Hunt–Hess scores 1–2 (*p* = 0.0073 and *p* = 0.0255). Two maximum values of PDK4 were removed from both the mild injury group and the Hunt–Hess scores 1–2 group.

### 3.8. Different Levels of PDK4 and Pyruvate Were Related to Long-Term Outcomes of SAH Patients

**Figure 8:**
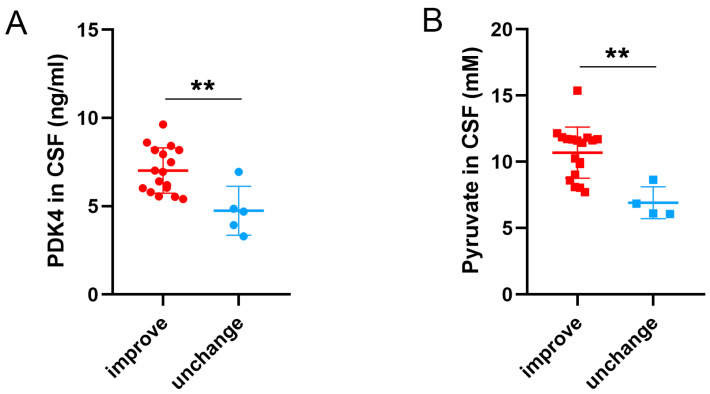
Scatter plots of the levels of PDK4 and pyruvate sorted by outcome category. Unpaired two-tailed *t*-tests indicated that higher expression of PDK4 and pyruvate was significantly associated with an improved outcome 3 months after SAH (**A**,**B**). Bars represent the means ± SD. ** *p* < 0.01 vs. the indicated groups (*n* = 5 in the unchanged group and *n* = 17 in the improved group).

To explore the correlation of PDK4 and pyruvate levels and long-term outcomes of SAH patients, the difference in GOS scores at 3 months compared with 1–3 days after SAH was used to assess the long-term outcome of SAH patients. Subjects whose initial GOS scores and GOS scores after 3 months were both 5 were excluded. As shown in Figure 8, the levels of PDK4 and pyruvate in the improved group were significantly higher than those in the unchanged group (*p* = 0.0027 and *p* = 0.0011). Two maximum values of PDK4 in the improved group were removed.

## 4. Discussion

In this study, we demonstrated that overexpression of PDK4 promoted the phosphorylation of PDH and inhibited PDH activity after SAH. The expression of PDH was unchanged, but the apoptosis signal-regulating kinase 1 (ASK1)/p38 pathway was activated after SAH, resulting in apoptosis. Previous studies suggested that activated PDH promotes the production of reactive oxygen species (ROS) [16]. ROS are highly reactive chemical molecules including peroxides, superoxide, hydroxyl radicals, and singlet oxygen [30]. Under normal conditions, ROS are present at low and stationary levels in cells and can participate in cell signaling and homeostasis [31,32]. However, ROS accumulate after pathological stimulation and cause irreversible damage to DNA. SAH damages the balance between ROS production and disposal and results in significant damage to cell structures during EBI. Therefore, early overexpression of PDK4 may protect cells from damage caused by ROS.

In our past research, we reported that the ROS-ASK1-p38 pathway promotes apoptosis after SAH [33,34]. ASK1, known as mitogen-activated protein kinase 5 (MAPK5), is a member of the MAP kinase family and activates c-Jun N-terminal kinase (JNK) and p38 mitogen-activated protein kinases in response to stresses such as oxidative stress, endoplasmic reticulum stress and calcium influx. In a variety of studies, ASK1 has been found to play an important role [35,36]. p38 is also a member of the class of MAP kinases and is responsive to stress stimuli [37,38]. Under pathological stresses, the activity of p38 is stimulated, and the activity of the transcription factor NF-κB is high. Dysregulation of p38 and NF-κB can result in apoptosis, autophagy, and inflammation [39,40]. Caspase3 plays a central role in apoptosis, and we found that activated caspase3 was significantly increased after SAH in vivo and in vitro [41]. Similarly, the apoptosis promotor Bax and inhibitor Bcl2 were upregulated by pathological stress after SAH in vivo and in vitro. Their interaction determines whether apoptosis occurs [42,43]. The early overexpression of PDK4 after SAH inhibited the activity of PDH, promoted the efficient elimination of ROS and influenced the activity of the ASK1/p38 pathway, caspase3, Bax, and Bcl2. Therefore, we hypothesized that PDK4 could protect cells, lessen neurofunctional injuries and function as a biomarker of outcome prediction after SAH.

We detected the levels of PDK4 and pyruvate in the CSF of SAH patients and controls, and the results showed that the levels of PDK4 and pyruvate were significantly elevated in the CSF of SAH patients compared with controls. Pyruvate was the reaction substrate of PDH and represented the activity of PDH. In some studies, the accumulation of pyruvate can protect cells from ROS damage [18]. GCS scores and Hunt–Hess Scale scores 1–3 days after SAH were used to assess SAH severity, and GOS scores at 3 months compared with 1–3 days after SAH were used to assess the long-term outcome of SAH patients [44,45]. After analyzing PDK4 and pyruvate levels in the CSF of SAH patients, we found that patients with mild injury had higher levels of PDK4 and pyruvate in CSF and that there was a significant correlation between the levels of PDK4 and pyruvate in CSF and the long-term outcome of SAH patients.

To the best of our knowledge, this is the first report to demonstrate that early overexpression of PDK4 inhibited the activity of PDH and may play a protective role after SAH. Furthermore, we found that PDK4 and pyruvate were acutely elevated in the CSF of SAH patients compared with controls, and their high expression correlated with the degree of neurological impairment and long-term outcome. This is clinically meaningful and could function as a new therapeutic target or biomarker predicting initial injury to the brain and recovery potential. However, several limitations of this study should be addressed. The corresponding mechanisms should be explored further, and we plan to work toward this goal as the next step in our research. In addition, the number of subjects was relatively small, and thus we will continue to recruit patients for more reliable conclusions. As described above, we explored the expression variation and function of PDK4 in animal, cell, and clinical experiments. These results showed that PDK4 is worth further exploration and has potential value for clinical transformation, serving as a new therapeutic target and biomarker.

## 5. Conclusions

Early overexpression of PDK4 promoted the phosphorylation of PDH and inhibited PDH activity after SAH. PDK4 has the potential to serve as a new therapeutic target and biomarker for use in the diagnosis of SAH severity and the prediction of recovery.

## Figures and Tables

**Table 1:** Summary of demographic data in patients with SAH.

Case No.	Age (Years)	Gender	Hyper-Tension	Aneurysm	Days after Hemorrhage (d)	Initial GCS	Initial Hunt-Hess	Initial GOS	GOS (after 3 Months)
1	63	F	1	1	1	15	3	5	5
2	72	F	1	1	1	15	1	5	5
3	54	M	0	1	1	3	4	1	1
4	75	F	0	1	1	8	4	3	3
5	65	F	1	1	1	15	2	4	5
6	69	F	1	0	1	15	2	5	5
7	69	F	0	1	1	15	3	5	5
8	48	M	0	1	1	14	3	3	4
9	64	M	1	1	2	14	3	4	5
10	76	M	0	1	2	7	4	3	5
11	40	F	1	1	2	15	3	3	5
12	50	F	0	1	2	9	4	3	5
13	62	F	1	1	2	5	4	1	1
14	49	F	0	1	2	12	3	4	5
15	56	F	1	0	2	15	1	4	5
16	66	F	1	1	2	5	3	5	5
17	63	M	0	0	2	14	2	5	5
18	55	M	1	1	2	15	1	5	5
19	45	M	1	1	2	15	3	5	5
20	63	M	1	0	2	15	2	5	5
21	73	F	1	1	2	14	3	3	3
22	45	F	1	1	2	15	2	5	5
23	47	F	0	1	3	15	2	4	4
24	49	M	0	1	3	15	2	4	5
25	53	M	0	0	3	15	2	4	5
26	54	F	0	1	3	15	2	4	5
27	72	F	0	1	3	14	1	4	5
28	55	M	0	1	3	15	2	4	5
29	63	F	1	1	3	15	3	4	5
30	68	M	1	1	3	14	3	4	5
31	57	F	1	1	3	14	2	4	5
32	65	F	1	1	3	14	2	3	5
33	57	F	1	1	3	15	1	4	5
34	52	M	1	1	3	15	1	5	5

d, days; GCS, Glasgow Coma Scale; GOS, Glasgow outcome scale lines of hypertension and aneurysm; 0, No; 1, yes; F, female; M, male.

**Table 2:** Polymerase chain reaction (PCR) primer sequences.

Target Gene	Forward (5′ to 3′)	Reverse (5′ to 3′)
PDK1	GTTCAGTACTTTTTGGATCGGTTC	TCGACTACATCACAGTTTGGATTT
PDK2	TGGACCGCTTCTACCTCAG	TCTTTCACCACATCAGACACG
PDK3	TGACCTAGGTGGTGGAGTCCCA	ACCAAATCCAGCCAAGGGAGCA
PDK4	GAACACCCCTTCCGTCCAGCT	TGTGCCATCGTAGGGACCACA
BAX	GACACCTGAGCTGACCTTGG	GAGGAAGTCCAGTGTCCAGC
BCL2	TATGATAACCGGGAGATCGTGATC	GTGCAGATGCCGGTTCAGGTACTC
GAPDH	TGTGAAGCTCATTTCCTGGTA	TTACTCCTTGGAGGCCATGT

**Table 3:** Mortality and exclusion.

Groups	Mortality Rate	Excluded
Stage 1		
Sham	0 (0/5)	0
SAH 6 h	11% (1–9)	3
SAH 24 h	22% (2–9)	2
SAH 2 d	22% (2–9)	2
SAH 3 d	11% (1–9)	3
SAD 7 d	22% (2–9)	2
Stage 2		
sham for IF	0 (0/6)	0
SAH 2 d for IF	22% (2–9)	1
Total		
Sham	0 (0–11)	0
SAH	18.5% (10–54)	13

## Data Availability

The datasets supporting the conclusions of this article are included within the article.

## Abbreviations

acetyl-CoA, acetyl-coenzyme A; ASK1, apoptosis signal-regulating kinase 1; CSF, cerebrospinal fluid; GAPDH, glyceraldehyde 3-phosphate dehydrogenase; GCS, Glasgow Coma Scale; GFAP, glial fibrillary acidic protein; GOS, Glasgow outcome scale; EBI, early brain injury; Hb, hemoglobin; HRP, horseradish peroxidase; ICA, internal carotid artery; Iba1, ionized calcium binding adaptor molecule 1; JNK, Jun N-terminal kinase; MAPK5, mitogen-activated protein kinase 5; MCA, middle cerebral artery; MOMP, mitochondrial outer membrane permeabilization; NeuN, neuronal nuclei; PBS, phosphate-buffered saline; PDH, pyruvate dehydrogenase; PDK, pyruvate dehydrogenase kinase; PPAR, peroxisome proliferators-activated receptor; qPCR, real-time quantitative polymerase chain reaction; ROS, reactive oxygen species; SAH, subarachnoid hemorrhage; WB, western blotting.
