# Correction: Gao et al. High Expression of PDK4 Could Play a Potentially Protective Role by Attenuating Oxidative Stress after Subarachnoid Hemorrhage. *J. Clin. Med.* 2022, *11*, 3974

**DOI:** 10.3390/jcm13237269

**Published:** 2024-11-29

**Authors:** Xuan Gao, Yong-Yue Gao, Ling-Yun Wu, Zheng Peng, Xun-Zhi Liu, Xiang-Xin Chen, Sen Gao, Hua-Sheng Zhang, Yue Lu, Chun-Hua Hang, Zong Zhuang, Wei Li

**Affiliations:** 1 Department of Neurosurgery, Nanjing Drum Tower Hospital, The Affiliated Hospital of Nanjing University Medical School, Nanjing 210008, China; xuan_gao@126.com (X.G.); tallergao@163.com (Y.-Y.G.); dr.wulingyun@gmail.com (L.-Y.W.); neurosurgery_pz@163.com (Z.P.); liuxunzhi_surgery@163.com (X.-Z.L.); xiangxin.chen@foxmail.com (X.-X.C.); sen_gao@outlook.com (S.G.); njuzhs@163.com (H.-S.Z.); luyue120@nju.edu.cn (Y.L.); hang_neurosurgery@163.com (C.-H.H.); 2 Department of Neurosurgery, Tianjin Huanhu Hospital, Tianjin 300300, China

## Error in Figure

In the original publication [1], there were mistakes in Figures 3 and 4 as published. The fluorescent pictures of the SAH 2d group in Figure 3 and bands of the PDK4 and PDH groups in Figure 4 were wrongly chosen. The corrected figures appear below.

In Figure 3, the fluorescent pictures of the SAH 2d group have been corrected.

In Figure 4, the bands of the PDK4 and PDH groups have been corrected.

## Correct Email

Xun-Zhi Liu’s email address was changed from “5105197877@163.com” to “liuxunzhi_surgery@163.com”.

The authors state that the scientific conclusions are unaffected. This correction was approved by the Academic Editor. The original publication has also been updated.

## Figures and Tables

**Figure 3:**
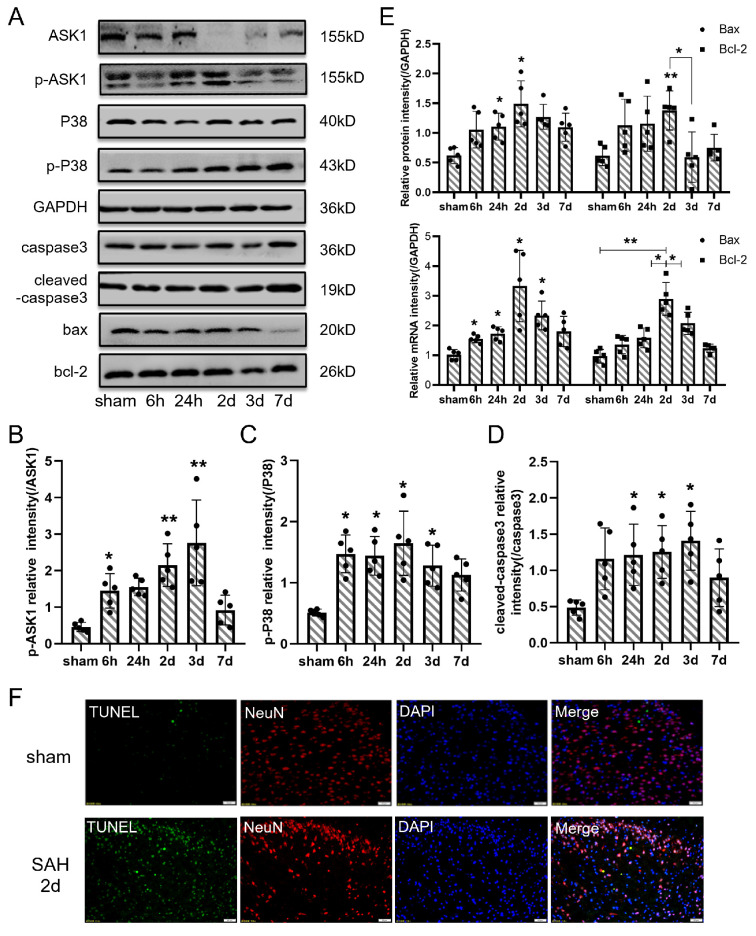
The apoptosis pathway was activated after SAH. (**A**) Representative bands of ASK1, p-ASK1, P38, p-P38, caspase3, cleaved-caspase3, Bax, and Bcl-2 expression in the cortex at each time point after SAH. (**B**–**D**) Quantitative analysis of Western blot results showed that the ratio of p-ASK1/ASK1, p-P38/P38 and cleaved-caspase3/caspase3 were significantly increased after SAH. (**E**) Quantitative analysis of Western blot and qPCR results showed that the protein and mRNA levels of Bax and Bcl-2 were increased after SAH. (**F**) Representative TUNEL staining in the cortex of right temporal lobe after SAH (TUNEL, green; NeuN, red; DAPI, blue). Bars represent the means ± SD. * *p* < 0.05; ** *p* < 0.01; vs. sham (*n* = 5 in each group). Bar = 50 μm.

**Figure 4:**
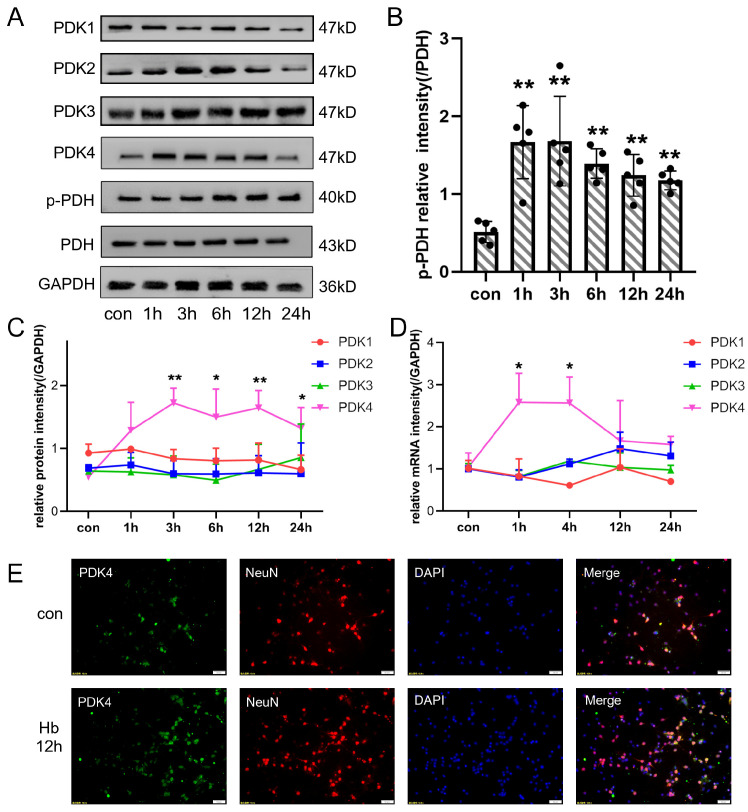
The expression of PDKs in cultured primary neurons after Hb stimulation. (**A**) Representative bands of PDK1, PDK2, PDK3, PDK4, PDH, and p-PDH expression at each time point (0, 1, 3, 6, 12, and 24 h) after Hb stimulation. (**B**) Quantitative analysis of Western blot results showed that the ratio of p-PDH/PDH was significantly increased after Hb stimulation. (**C**,**D**) Quantitative analysis of Western blot and qPCR results showed that variation of PDKs protein at each time point (0, 1, 3, 6, 12, and 24 h) and mRNA at each time point (0, 1, 4, 12, and 24 h) levels after Hb stimulation. (**E**) Representative immunofluorescence staining for PDK4 and NeuN (a neuronal marker) in cultured primary neurons after Hb stimulation (PDK4, green; NeuN, red; DAPI, blue). Bars represent the means ± SD. * *p* < 0.05; ** *p* < 0.01 vs. con (*n* = 5 in each group). Bar = 50 μm.
